# On Operations for the Results of Cholelithiasis

**Published:** 1910-02-19

**Authors:** 


### ON OPERATIONS FOR THE RESULTS OF CHOLELITHIASIS.

Gall-stones may be found in any part of the biliary apparatus from the gall-bladder itself to the opening of the common duct into the duodenum, known as "the ampulla of Vater." Reference is going to be made in this article only to operations performed for stones in the bladder. But no hard and fast line can be laid down, and for several reasons. First of all, stones may exist even for years in the bladder itself and give rise to few or no symptoms. It is generally only when one or more of them begin to travel that jaundice or colic or other phenomena associated with gall-stones make their appearance and the patient's attention is directed to his state of health. There are two corollaries to this statement: one is that every operation upon a patient presenting symptoms of cholelithiasis is, in a sense, an exploratory operation; and the other, as has been pointed out by Mr. Mayo Robson, that no surgeon is justified in operating upon such a case unless he is acquainted with the technique of the various operations which have been devised for this condition, and is prepared to perform any one of them should the occasion arise.

To expose the gall-bladder is a comparatively simple matter. One preliminary precaution must, however, be observed, and that is to place a sandbag under the right hepatic region before the operation is begun. The object of this is to throw the gall-bladder forwards, so that it will tend to present from under the surface of the liver when the abdomen is opened. It is astonishing what a difference this makes and how deep the gall-bladder may lie if the patient is merely placed upon a flat table in the supine position.

The incision which is in general use for this operation is a vertical one, four inches long, extending downwards from the top of the ninth right costal cartilage, the surface marking of the fundus of the gall-bladder. This will be found to correspond to the upper of the right linea semilunaris. If, as sometimes happens, more room is needed, the incision may be enlarged upwards and inwards parallel to and about one inch below the costal arch for a distance of two inches. The writer has seen one case in which it was necessary to remove a portion of the eighth and ninth right costal cartilages before the gall-bladder could be adequately exposed. But this must be rarely called for.

The ease with which the gall-bladder can be brought up into the wound varies in every case. Where the viscus is distended with stones, or even of normal size, the matter is simple; but it is the reverse of this if the viscus is shrunken and surrounded by adhesions resulting from old attacks of inflammation in its neighbourhood as sometimes occurs, and particularly if the liver itself is atrophic and its interior margin is retracted for some distance behind the costal arch. In such cases the adhesions must be patiently broken down and the peritoneal attachments of the gall-bladder to the liver divided before it can be exposed.

The next question which must be answered is, Should the gall-bladder be opened and drained after removal of the stones, or should it be removed entirely? It would appear not unreasonable to compare the gall-bladder to the appendix—and, indeed, in many respects the comparison is peculiarly apposite. One might argue, for instance: In the case of a chronically inflamed appendix which is full of concretions, in this case stercoliths, one removes the organ entirely. Why not the gall-bladder in the same way? This is surely the ideal operation. But, as a matter of fact, cholecystectomy is not an ideal routine operation, as Mr. Mayo Robson has pointed out. The technique is more difficult than that of simple cholecystostomy, and is liable to be followed, except in the hands of the most experienced operators, by a higher mortality; and, further, it has been shown that when the bladder has been removed, the common duct may subsequently dilate and form an emergency gall-bladder in which stones may reaccumulate. There are, of course, certain positive indications for cholecystectomy, *i.e.* where it would endanger the life of the patient to leave the organ *in situ*. They are (i.) any form of malignant disease of the organ, (ii.) acute inflammatory conditions, with or without the presence of pus, which may set up a fatal peritonitis if left, and (iii.) an advanced contraction of the bladder which is incapable of performing its functions as the result of multiple past attacks of inflammation.

When the gall-bladder contains calculi and none of these indications is present, cholecystostomy is an easier and more satisfactory procedure as a routine measure. The bladder is opened by incising its fundus, after packing it round with gauze to prevent contamination of the adjacent peritoneum. The stones are removed with a scoop. After emptying the bladder the duct must be explored, in case a single calculus has become impacted in it. The most satisfactory way of doing this is to pass a probe gently down it with the left hand, while the right index finger is passed down along the outside of the duct. When all the stones have been removed, a rubber tube should be inserted into the bladder and fixed in with a purse-string suture. The gall-bladder is then anchored to the parietal peritoneum and posterior layer of the rectus sheath by interrupted silk stitches, which take up the covering of the gall-bladder only, and the rest of the wound is closed in the ordinary way. It is convenient to leave the tube sufficiently long to protrude through the dressings, and a thin rubber tube, such as is used with a Paul's tube, can be applied to it, so that drainage can take place into a porringer placed at the side of the bed without disturbing the dressings.

---

**Pityriasis Versicolor.**

This affection may be promptly cured by turpentine; the clothes must also be disinfected.

**Syphilis and the Nervous System.**

The sooner the nervous affection follows the primary infection, the more likely is it to be a syphilitic disease of the nervous system; the longer the interval the more probable is parasyphilitic disease. It is rare for generalised syphilitic meningitis to commence after five years—one half the cases occur in the first eighteen months. It is also rare for tabes dorsalis or G.P.I. to arise within a few years after infection.—*Dr. F. W. Mott.*

**The Induction of Sleep.**

The instant the mind is brought to the contemplation of a single sensation, that instant the sensorium abdicates the throne, and the hypnotic faculty steeps it in oblivion. Having arranged his head comfortably on the pillow, the patient takes a very full inspiration, and then the lungs are to be left to their own action. But the patient must now depict to himself that he sees the breath passing from his nostrils in a continuous stream, and the moment he brings his mind to conceive this, apart from all other ideas, he sleeps.—*Dr. Pereira.*

